# One size fits all? On the institutionalization of participatory technology assessment and its interconnection with national ways of policy-making: the cases of Switzerland and Austria

**DOI:** 10.1007/s10202-012-0120-7

**Published:** 2012-11-16

**Authors:** Erich Griessler

**Affiliations:** Department of Sociology, Institute for Advanced Studies, Stumpergasse 56, 1060 Vienna, Austria

## Abstract

Science and technology policy is often confronted with issues that are both complex and controversial and which have to be decided upon in a delicate constellation of policy-makers, experts, stakeholders, non-governmental organizations and the public. One attempt to deal with such a complex problem is via citizen involvement. Participatory technology assessment (pTA) already goes back to several decades, and countries have made various experiences. While in some countries, governments established technology assessment organizations, which also included pTA in their methodological portfolio, others primarily rely on experts to make decisions on science and technology policy. In a third group of countries non-state actors, such as social scientists, experimented with pTA. However, they were often unable to link these experiments to policy-making. This paper deals with the question of why this variation exists and compares the use of pTA in Switzerland and Austria. Despite similarities between the two countries, both had quite different experiences with pTA so far. Whereas several pTAs have been carried out in Switzerland until today, Austrian pTAs have remained infrequent. The aim of this paper is to explain this difference as a result of different ways of policy-making which affect the use and chances of pTA.

## Abstract (de)

Forschungs- und Technologiepolitik ist oft mit Themen konfrontiert, die sowohl wissensintensiv als auch kontrovers sind und in einer schwierigen Konstellation von EntscheidungsträgerInnen, ExpertInnen, Stakeholdern, Nicht-Regierungsorganisationen und der Öffentlichkeit entschieden werden müssen. Ein Ansatz, mit solchen komplexen Problemen umzugehen, sind Verfahren der Bevölkerungsbeteiligung. Die Geschichte des Participatory Technology Assessments (pTA) reicht bereits einige Jahrzehnte zurück und die Erfahrungen, die damit in verschiedenen Ländern gemacht wurden, sind durchaus unterschiedlich. Während Regierungen in einigen Ländern Organisationen eingerichtet haben, deren methodisches Portfolio der Technikfolgenabschätzung auch pTA-Ansätze mit einschließt, vertrauen EntscheidungsträgerInnen in anderen Staaten bei Technikfolgenabschätzung vor allem auf ExpertInnen. In einer dritten Gruppe von Ländern haben nicht-staatliche AkteurInnen, wie zum Beispiel SozialwissenschaftlerInnen, auf eigene Initiative damit begonnen, mit pTA zu experimentieren, waren jedoch häufig nicht in der Lage, diese Experimente effektiv an politische Entscheidungsprozesse zu koppeln. Dieser Beitrag beschäftigt sich mit dem Problem, warum diese länderspezifischen Unterschiede in der Anwendung von pTA existieren und versucht diese Fragestellung mit unterschiedlichen Praktiken des Politikmachens zu erklären. Der Artikel vergleicht dazu Österreich und die Schweiz. Während in der Schweiz eine Reihe von pTAs erfolgreich durchgeführt wurden, blieb in Österreich die Zahl an Bevölkerungsbeteiligungsverfahren im Bereich von Forschung und Technologie gering.

## Résumé

La politique de recherche et technologique se voit souvent confrontée à des problèmes demandant beaucoup de connaissance et suscitant des polémiques et sur lesquels décideurs, experts, parties prenantes, organisations non-gouvernementales et le public doivent décider dans des constellations difficiles. Un moyen pour surmonter ces difficultés, ce sont les procédés de participation de la population dans les décisions qui affectent leur vie. L’histoire de l’évaluation participative des conséquences technologiques remonte à quelques décennies déjà, et les expériences acquises dans différents pays sont assez divergents. Tandis que dans certains pays, les gouvernements ont établi des organisations dont le portfolio méthodique d’évaluation des conséquences technologiques incluait aussi des approches d’évaluation participative, dans d’autres pays, les décideurs s’appuient en premier lieu sur les connaissances des experts. Dans un troisième groupe de pays, des acteurs non-étatiques tels que par exemple des spécialistes des sciences sociales, ont fait leurs propres expériences dans l’évaluation participative des conséquences technologiques. Mais souvent ces expériences n’ont pas été prises en compte dans les processus décisionnels politiques. Pourquoi y a-t-il ces différences spécifiques aux pays dans l’évaluation participative des conséquences technologiques? Le présent article tente d’expliquer ce problème par les différentes pratiques d’élaboration de politiques en comparant l’Autriche à la Suisse. Tandis qu’en Suisse, nombre d’évaluations participatives des conséquences technologiques ont été réalisées avec succès, le nombre de procédés de participation de la population en Autriche est resté négligeable.

## Introduction

Science and technology policy is often confronted with issues that are both knowledge intensive and controversial. Frequently cited examples include genetically modified organisms (GMOs), nuclear energy and nanotechnologies. Such complex issues have to be decided upon in a delicate constellation of policy-makers, experts, stakeholders, NGOs and the public; in other words, within relationships that are often laden with controversy and conflict. One attempt to disentangle this Gordic knot is via citizen involvement. Participatory technology assessment (pTA), that is, a structured process to include the public in decision making, already goes back several decades and countries have so far had varied experiences (Joss and Bellucci 2002). While in some countries governments established technology assessment organizations, which also included pTA in their methodological portfolio, others solely rely on experts to make decisions on science and technology policy. In a third group of countries, non-state actors, such as social scientists, tried to remedy the absence of pTA procedures in official policy-making and took initiative to experiment with pTA methods. However, they were often unable to link these experiments to policy-making.

This paper addresses the question, why this variation exists. Why were some countries successful in introducing pTA into decision making on science and technology policy, while similar attempts remained unsuccessful in others? In order to address this question, the paper compares the experiences in Switzerland and Austria. The two neighbouring countries are small, Western European democracies, and—in the case of Switzerland, partly—share one language. Both have well-established Technology Assessment (TA) institutions, which are linked to their respective Academy of Sciences. It would therefore be fair to assume that they share great similarities in the use of pTA. However, both countries had quite different experiences. Whereas several pTAs have been carried out in Switzerland so far (Pfersdorf 2008; Crettaz von Roten 2011), Austrian pTAs have remained infrequent (Grabner et al. 2002; Bogner 2004; Nentwich et al. 2006; Felt et al. 2006; Degelsegger and Torgersen 2011).

The main aim of this paper is to explain this difference as resulting from different types of policy-making by means of which pTA is approached in the two countries. The paper will therefore sketch out different practices of policy-making as well as roles played and self-conceptions adopted by politicians, civil servants, experts and citizens as expressed therein. It will do this by using the example of xenotransplantation, which is the transplantation of cells, tissues and organs across species. In the late 1990s, xenotransplantation was perceived as constituting a promising technology but also as being connected to a number of risks and ethical problems; it therefore became a matter of regulation. Taking the regulation of xenotransplantation as an example, the paper looks at how policies were developed in Switzerland and Austria, which actors where involved, and what role pTA played. Since there was little policy-making in Austria in xenotransplantation, the paper takes a wider perspective by looking at pTA in Austria more generally and different cases of policy-making in science and technology policy in the life sciences.

## Methods

The Swiss case is based on eleven in-depth, face-to-face interviews with experts from TA-organizations, xenotransplantation research, public administration, politics and bioethics. The interview partners were either involved in xenotransplantation research, TA and pTA on xenotransplantation and/or the respective policy-making process. The interviews were carried out in 2010. They were taped, fully transcribed and analysed using ATLAS.ti, a software package for qualitative content analysis. Data analysis followed Meuser and Nagel’s (2002) suggestions for analysing expert interviews and focussed entirely on the manifest content.^1^ Beyond this, the Swiss case study includes an analysis of material from the Fraunhofer Institute for Systems and Innovation Research (FhG-ISI), generated during their TA studies on xenotransplantation in the late 1990s. Moreover, analysis covers documents and publications provided by TA-Swiss on the TA and pTA on xenotransplantation, materials produced during the law-making process (e.g. draft bills, reports, records) and newspaper articles provided either by interview partners or retrieved from the Internet.

The Austrian case draws on previous research on the regulation of various innovative areas of biomedicine such as transplantation and xenotransplantation, human embryonic stem cell research and pre-implantation genetic diagnostics (Griessler 2010), genetic testing (Griessler and Lehner 2010) and prenatal diagnostics (Griessler 2012). Also in this case expert, interviews with politicians, civil servants, experts and other stakeholders were carried out and policy documents as well as newspaper articles were analysed.

## Switzerland

### Xenotransplantation policies

The following section provides a short overview on the development of xenotransplantation policies in Switzerland.

Xenotransplantation became an issue of public debate in Switzerland in the 1990s. However, the debate was neither extensive nor continuous and only of concern to a small group of informed actors from research, parliament, public administration and stakeholder organizations. Public interest eventually faded when xenotransplantation research did not advance as rapidly as predicted.

The debate was controversial and the positions taken on the matter ranged from clear approval to strict rejection (Bellucci et al. 1998: 157; Hüsing et al. 1998: 172 ff.). Supporters argued that xenotransplantation could alleviate organ shortage, contribute to saving patients’ lives and increase their quality of life. Responding to animal welfare arguments, they emphasized that each year millions of pigs would be slaughtered in Switzerland for food anyway (8: 273–287) and that using animals for medical purposes constituted a far higher goal than eating them (4: 71–81).

Opponents introduced aspects of animal welfare into the debate and voiced concerns about potential infection risks. They questioned patients’ survival rates, the quality of life of recipients and raised possible problems related to patient’s sense of identity and psychological states (2: 155–164, Hüsing et al. 1998). Moreover, they questioned whether it would be ethically acceptable to genetically modify animals. They argued that xenotransplantation would basically result in spectacular profits for the pharmaceutical industry.

In 1996, the biotechnology critical NGO *Basler Appell gegen Gentechnologie,*^2^ the Swiss Parliament, the Federal Office for Public Health (*Bundesamt für Gesundheit,* FOPH) and TA-Swiss became aware of xenotransplantation (Griessler 2011). This activity was partly triggered by a British market study which forecasted a huge increase in xenotransplantation in the near future (Laing 1996). Already in the same year, TA-Swiss commissioned a study on xenotransplantation.

In 1996 and 1997, initiatives to regulate xenotransplantation reached the Swiss Parliament. In 1999, its two Chambers were divided whether to follow a restrictive governmental bill and ban the application of xenotransplantation in principle, allowing only for exceptions. In Parliament, xenotransplantation was primarily discussed in the context of research and economic policy. Members of Parliament from a majority of conservative and liberal parties emphasized the importance of the pharmaceutical industry for Swiss research and Switzerland as a business location. They opposed a ban or moratorium with the argument that Switzerland could not stand alone with such a political measure. Highlighting the promises of xenotransplantation, they advocated research into xenotransplantation with prior authorization. Finally, the Parliament adopted a permissive law which allowed xenotransplantation but made it subject to requirements and authorization.

In 2000, this approach was also approved by the majority of lay participants at a consensus conference type “PubliForum” on transplantation and xenotransplantation, which TA-Swiss organized in cooperation with the responsible ministry and the Swiss Academy of Science.

### (Participatory) technology assessment

TA-Swiss, a small TA organization that was still linked to the Swiss Parliament at the time, perceived xenotransplantation as an up and coming biomedical development, the social and ethical impact of which required closer investigation. It therefore initiated two expert studies (Hüsing et al. 1998, 2001) to get an overview of the field and its implications for Switzerland (Schweizerischer Wissenschaftsrat 1996a, b; Bellucci et al. 1998).

TA-Swiss first contracted a TA study on solid organ xenotransplantation, which followed a classical TA approach in one sense. The study was to provide an overview on the international state of the art and its positive and negative consequences from societal, ethical-anthropological, economic and clinical perspectives. It set out to investigate the Swiss situation with regard to acceptance of xenotransplantation and regulatory questions (Schweizerischer Wissenschaftsrat 1996a). On the other hand, the study involved elements of invited participation (Hüsing et al. 1998: 9 ff.; 231); it included in-depth interviews with Swiss experts and an open and qualitative survey, in which organized stakeholders were asked for statements on xenotransplantation (Hüsing et al. 1998: 232; Bellucci et al. 1998: 157). The study was published in August 1998 and took a cautious approach towards xenotransplantation. This study was followed by a TA on cellular xenotransplantation (Hüsing et al. 2001).

Based on the Danish consensus conference model, TA-Swiss subsequently organized a PubliForum on transplantation, which also addressed xenotransplantation (Zentrum für Technologiefolgen - Abschätzung et al. 2001). The PubliForum involved 28 citizens who were selected to represent the Swiss population. The citizens had significant influence on framing the issue under discussion by raising the questions they wanted to discuss and inviting the experts they wanted to listen to. Supported by TA-Swiss and a facilitator, participants formulated a report, which they presented to Members of Parliament, the responsible ministry and The Swiss Academy of Science. The outcome of the PubliForum was therefore well connected with established policy-makers. However, it seems the results of the PubiForum were not disseminated as widely as to reach the general public.

The expert TA and PubliForum were well received by the political system and xenotransplantation researchers. The TA study was discussed in Parliament and its quality was recognized in the parliamentary debate. It was extensively referred to in documents of the responsible ministry and in the message to Parliament. The opinion of the PubliForum with regard to xenotransplantation was also shared by the ministry and Parliament.

The PubliForum on transplantation was not the first and last pTA that TA-Swiss organized. Participatory forms of TA became a standard procedure, which is, however, not used in all of its studies. Besides traditional expert TA, participatory instruments used by TA-Swiss also include shorter versions of public participation such as PubliTalk and PubliFocus.

Swiss xenotransplantation policy cannot be directly linked to the outcome of the pTA because the main decisions were made before the PubliForum. Moreover, the majority of the PubliForum supported government policy. The PubliForum therefore mainly had a legitimizing impact, since the recommendation corresponded with and supported government policy.

### Policy-making

The following section focuses on central characteristics and institutions of policy-making in Switzerland and draws on literature about its political system (Linder 1999a, b, 2009; Lüthi 1999) and TA in Switzerland (Pfersdorf 2008).

#### Concordance

Switzerland is the model of consensus democracy, which involves federal structure, concordance and power sharing. Power sharing is based on federalism, subsidiarity and voluntary proportional representation in assigning different political offices with regard to language, party affiliation and gender (Linder 1999a: 467 ff.). The concordance system developed over decades; this development began in the mid-nineteenth century out of a political conflict involving the dominant Liberals and in which different political movements struggled for political power and were subsequently included in government (i.e. Catholic-Conservatives in 1891; Farmers-, Trading- and Citizens-Party after World War I and finally the Social Democrats only in 1959) (Linder 1999a: 468, b: 14ff).

The concordance system can be explained against the background of direct democracy, which poses a continuous threat to decisions made in the representative system and therefore acts as a strong coercion towards concordance. In other words, to involve the main political actors considerably reduces the risk of government being defeated by plebiscite.

#### Federalism

Switzerland is a state with strong federalist elements. The Federation and the 26 cantons have their own constitutions and the around 3,000 communities enjoy large autonomy. Federalism was important for the maintenance of a peaceful coexistence of different ethnicities and religious groups. The implementation of the concept of subsidiarity turns Switzerland into one of the most decentralized countries (Linder 1999a: 457).

#### Direct democracy

Citizen participation is particularly rich in Switzerland, and direct democracy plays a central role in the political system. Instruments include (1) obligatory constitutional referendum, (2) facultative referendum on laws, (3) people’s initiative. On average, the electorate has to vote on six constitutional changes and two to four referenda on laws annually (Linder 1999a: 462 ff.).

Swiss direct democracy gradually developed out of a nineteenth century grassroots movement, which distrusted representative democracy and wanted to limit the power of parliament (Linder 1999a: 463). Though elites play a strong role in the political system, the electorate can also play an active role by means of direct democracy. By threatening the political elite with loss of power and reputation, voters can force a reaction; they therefore constitute a control factor when it comes to important questions and are significant veto players. The risk that a referendum might overturn a law is always present; for this reason, “the entire Swiss political system is intended to avoid a referendum” (9: 300). The Swiss system is also referred to as a “half-direct-democracy” because of the checks and balances between government (Federal Council, *Bundesrat*), Parliament and the electorate. In other words, these take place between representative democratic and direct democratic elements, which should enable co-determination of the voters on not all but the most important matters (Linder 1999a:463).

#### Government

The Swiss political system is located between a presidential and parliamentary system. The government consists of seven members. The two chambers of Parliament elect one member from the Federal Council as head of state annually (Federal President, *Bundespräsident*), who only has coordinative functions.

Government plays a strong and independent role vis-á-vis Parliament. Though Parliament often asks government to take regulatory steps by means of so-called *Motions,* it is the Federal Council that typically prepares laws and plays a main role in law-making. In reality, the position of the Swiss Government is close to a presidential system.

Federal Councillors (*Bundesräte*) are elected for four-year terms and cannot be removed from their posts, whereas re-election is possible. The composition of the Federal Council acknowledges the country’s multi-ethnicity as well as the strength of political parties from a long-term perspective. Since 1959, the Federal Council is composed of representatives of the four major parties. Until 2003, the so-called magical formula *(Zauberformel)* stipulated how seats are to be allocated to parties. The rule of proportional representation of language groups and parties is a major characteristic of Swiss concordance democracy; it is not laid down in the constitution but is a tradition followed by political actors voluntarily (Linder 1999a: 459–460). Parties adhere to this very slowly changing distribution of power, which is not dependent on the latest election results. Since the 2003 elections, which made the conservative populist SVP the strongest party, the concordance system is under pressure but is nevertheless still in place after some turbulences with only some slight modifications (Linder 2009: 571, 573, 597).

#### Parliament

Though Parliament is the highest political institution, its competences are delimited by the strong role of government, the strong elements of direct democracy and the involvement of interest organizations. The Swiss Parliament is a working parliament but, in comparison with the executive, has a much smaller infrastructure. It has the right to initiate and pass laws and budget, financial planning as well as to supervise government and administration. Its two chambers, the National Council *(Nationalrat,* 200 seats) and the Council of States *(Ständerat,* 46 seats), are equally important. In order to pass, a bill has to find a majority in both chambers. In cases of disagreement between the two chambers—which both deal with each bill separately and in detail—a procedure is launched to settle the difference. If these negotiations do not succeed, the law fails (Linder 1999a: 461ff.).

Swiss parliamentarians are working in the so-called Milizsystem, that is, they have a civilian profession and their political mandate is considered an additional occupation. Members of Parliament are not controlled by party discipline, must not be given instructions and are only responsible to their conscience. Parties therefore play a less important role than in many other western democracies.

The Swiss Parliament plays a strong formal role in legislation. It can refuse a governmental bill without discussing its content and can make detailed changes. By parliamentary initiatives, it can also make laws without the involvement of government and administration. It has a question right *(Anfrage)* and the right to request a response to a question *(Interpellation)* from government. Moreover, there are the instruments of *Postulat* in which Parliament asks government to examine whether it would be possible to draft a law and the *Motion,* which orders a governmental bill. Due to the strong role played by the government in the Swiss political system, the political majority in Parliament is less concerned with retaining the power of government and can play an independent and strong role as well (Linder 1999a: 462).

Switzerland features a relatively small bureaucracy (Linder 1999a: 476). Public administration plays an important role in law-making, since expert knowledge and experience in the implementation of laws accumulates in the civil service (Linder 1999a: 469). Bills are in most cases prepared by the Federal Council and administration (94.2 % of all laws between 1991 and 1995, Lüthi 1999: 139). The influence of the parliament on legislation is perceived differently in the literature. While some authors only attribute a marginal role to the legislature and instead emphasize the importance of the government and public administration, other authors call attention to the fact that government drafts are often modified during parliamentary negotiations (Lüthi 1999: 142).

#### Law-making

Typically government prepares a draft law and sends it into a formal consultation process *(Vernehmlassungsverfahren,* see Linder 1999a: 470), in which stakeholders can express their opinions on a bill and propose changes. The consultation helps to avoid referenda, which might result in a defeat of government by voters. The call for a consultation process is published. If the responsible ministry considers opposition by powerful actors as too strong, it redrafts the governmental bill. After consultation and amendment, the bill is sent to Parliament as a so-called message *(Botschaft)*. The message is a comprehensive text, which includes the bill and background information on the proposal. Only then does Parliament start the process of discussing and formulating the law within its respective commissions.

#### Features of the political system

As described, the Swiss political system is characterized by a division and an interconnection of power between the executive and legislative but also by the autonomy of cantons, interest groups, parties and single institutions such as the government. All of these are proportionally represented in decision-making processes and have veto power. Switzerland is therefore a model case of a *concordance democracy.* Advantages of this system include the integration of interest groups, the protection of minorities and that solutions find broad acceptance. Disadvantages include lengthy decision-making processes, a strong notion of elite cooperation and a lack of transparency to outsiders of the system (Pfersdorf 2008).

One of the main features of the Swiss political system is its orientation towards *consensus.* The constitution lays down the principle of collegiality in government, that is, the Federal Council has to find a common policy. The magic formula and the consultation process also express an orientation towards consensus.

Another characteristic of the Swiss political system identified by an interview partner is its *general pragmatism,* in the sense of concentrating on given circumstances. This attitude was expressed in the context of xenotransplantation policies in the sense that animal welfare issues and the infection risk were taken seriously and were pragmatically integrated into the regulation by stating that organs had to be tested prior to transplantation. The fundamental question, however, of whether xenotransplantation should be further pursued at all as a technology, was hardly debated from this pragmatic perspective (3: 138–177).

### pTA in the context of Swiss policy-making

As previously mentioned, *subtlebalances* between four language groups, cantonal and federal competencies and different political parties characterize the Swiss political system. There are tensions between representative democracy and direct democracy as well as a strong emphasis on an independent civil society. There is also a strong notion of *compromise* in ensuring that a shared community is sustained. Compromises are facilitated by a political discourse with a particular notion of *matter*-*of*-*factness,pragmatism* and, at least in the context of xenotransplantation, avoidance of radical stances.

Taking into account the main characteristics of Swiss policy-making, pTA is therefore well suited and serves several of its previously mentioned basic needs, that is, a need for matter-of-fact and sober discussion by ordinary, independent citizens so as to inform policy-makers, who in a fragile and polycentric political system are constantly threatened by popular veto. In the opinion of one researcher, pTA suits direct democracy since it provides new information, discussion and, in contrast to newspapers, does not remain on a polemic level (8: 375–389). In this sense, a pTA such as the PubliForum can be interpreted as yet another mechanism within Swiss *concordance democracy* (9: 348–351).

Interviews showed that pTA, such as a PubliForum, is particularly well suited to and complements existing channels, through which the Swiss electorate participates in legislation and political decision making; a *consultation* is only carried out in the context of legislation. Moreover, in reality, the consultation mechanism is geared towards involving organized political actors. The right to initiative is a way for citizens to become active but is always geared towards constitutional changes. A referendum always occurs post-festum, when a law has been passed and the electorate can vote on it. In contrast, the PubliForum provides a means for the executive to actively approach a group of citizens, composed as much as possible of representatives of the population, and to invite them to voice their opinion on a topic. Since it is open to regular citizens, it is a much more informal and low-threshold approach than a consultation because ordinary citizens are often not conscious of their right to voice their opinion in a consultation. This consultative instrument is mostly used by established political organizations and authorities. On the other hand, it seems that the pTA is more directed at policy-makers than the general public, since it was noticed more by policy-makers than by the media and was relatively well integrated into the political decision-making processes.

However, it should be kept in mind that neither TA nor pTA is uncontested in the Swiss political system (4: 327–344, Crettaz von Roten 2011: 18). pTA had to face criticism from political parties advocating a lean state and glorifying the role of individual citizens and the electorate.

## Austria

### Xenotransplantation policies

While policy-makers in other countries and international organizations discussed xenotransplantation policies at the turn of the millennium, Austrian policy-makers decided not to take regulatory measures at the time and in 2004 transposed the relevant European Directive (2001/20/EC) into national legislation without there being much public discussion. This decision for regulatory inactivity around the turn of the millennium was not caused by a lack of information. Austrian civil servants were well informed about xenotransplantation, and some of them were rather sceptical because of the infection risk (Griessler and Littig 2006). They frequently participated in international conferences and workshops that dealt with regulatory problems of xenotransplantation. However, this involvement did not result in any broader debate on xenotransplantation policies because civil servants thought it would be pointless to start a discussion over a technology, which might perhaps become a reality in 10–20 years (D: 370–372). This matches a well-known strategy in Austrian policy-making, that is, postponing decisions in order to avoid debates (Griessler 2010: 165). The topic of xenotransplantation never left the realm of ministries and neither Parliament nor the broader public discussed the issue in any detail. Also, the scientific advisory system was almost entirely absent in Austrian xenotransplantation policies.

### Participatory technology assessment

Austrian policy-makers regularly emphasize public involvement and dialogue when they address problems of new technologies (Kronberger and Kerbe 2010: 19). Statements of the Austrian Council for Research and Technology Development (*Rat für Forschung und Technologieentwicklung,* in the following RFT; RFT 2003, 2009) exemplify this participatory language. However, even the RFT recognizes a discrepancy between rhetoric and reality and concludes that “selling science” is clearly dominant in Austrian science communication (RFT 2009: 29).

This raises the question to what extent the public is actually involved in Austrian science and technology policy and what the chances of pTA are. In this context, Degelsegger and Torgersen (2011) emphasize the significance of “deeply held beliefs and engrained practices of political decision-making” (391). Drawing on political practices in the regulation of various innovative areas of biomedicine, such as transplantation, xenotransplantation, human embryonic stem cell research, pre-implantation genetic diagnostics (PGD) (Griessler 2010), genetic testing (Griessler and Lehner 2010) and prenatal diagnostics (PND, Griessler 2012), it will be argued that pTA faces serious difficulties in Austria because of dominant political practices and assumptions about policy-making and the public.

Formalized procedures of citizen involvement in priority setting in science and technology policy and technology assessment are non-existent in Austria (Kronberger and Kerbe 2010: 17ff.). pTA was only adopted in the mid-1990s, and researchers agree that pTA exercises remained infrequent (Grabner et al. 2002; Bogner 2004; Nentwich et al. 2006; Felt et al. 2006; Degelsegger and Torgersen 2011). pTA processes were methodologically diverse and included mediation processes (Grabner et al. 2002), consensus conferences (Bogner 2004; Grabner et al. 2002: 61 ff., Felt et al. 2006: 116 ff.), stakeholder dialogues (Griessler and Littig 2006) and public discussions (Felt et al. 2003: 118 ff.). Issues discussed were, for example, traffic, picking research areas for public funding, ground level ozone, xenotransplantation and genetic testing. Evaluations of the success of Austrian pTA processes were diverse, and judgments range from regarding them as a failure to being partially successful. The problems encountered are similar to other countries and include inadequate financing and lack of time; problems in recruiting participants; unfavourable group dynamics in the lay panel; communication difficulties between experts and laypeople; insufficient connection to the general public and to political decision making (Joss and Torgersen 2002: 176; Felt et al. 2006). As Bogner (2010) puts it, pTA exercises in Austria remained “laboratory experiments”, insofar as they had no impact on political decision making whatsoever and were actually cases of top-down and invited participation, organized as methodological experiments by social scientists with no impact on political decision making whatsoever (Kronberger and Kerbe 2010: 17).

The following section analyses some of the reasons for this limited use of participatory exercises in science and technology policy.

### Policy-making

There is little discussion in the Austrian Parliament about science and technology. Topics such as human embryonic stem cell research (Griessler 2010), PGD (Griessler and Lehner 2010), genetic testing (Griessler and Pichelstorfer 2010) and xenotransplantation (Griessler and Littig 2006), which have been debated in Parliament in many other countries, were hardly discussed in Austria’s National Council. Though relevant laws obviously have to pass the Parliament for constitutional reasons, the involvement of Parliament in actual decision making is often limited. Like many parliamentary systems (Cruz-Castro and Sanz-Menéndez 2004: 113) and despite the theory of separation of power, laws in Austria are not prepared by the legislative. Instead law-making is concentrated in the executive branch, more precisely in the ministerial bureaucracy, where federal laws are typically prepared by the civil service (Tàlos and Kittel 2001) and enter Parliament as governmental bills *(Regierungsvorlage;* Pelinka 2008).

#### Civil servants

Civil servants prepare governmental bills either on assignment by their political superiors or on their own initiative (Griessler and Lehner 2010). They start their work with a first draft, which is negotiated in a number of phases involving a number of actors. During the first informal pre-consultation phase *(Vorbegutachtungsverfahren)*, the actors include civil servants from their own ministry and other ministries as well as important political actors who might be affected by the law, such as politically powerful special interest groups.

After civil servants consult political superiors in their ministry and amend the draft accordingly, the draft bill enters the official consultation phase *(Begutachtungsverfahren)*, in which other ministries, provincial governments, social partners and other potentially affected organized interest groups, and sometimes—because of particular expertise—individuals, are officially invited to comment on the draft bill. Civil servants then collect and analyse these comments. The draft bill is changed—often only marginally, but sometimes even substantially—before it enters the Council of Ministers. It has to be unanimously approved there to become a governmental bill, which is then sent to Parliament. During the ensuing parliamentary process, it is the central goal of the ministry in charge to get the law through Parliament “undamaged” (Griessler and Lehner 2010: 130).

Several political practices of civil servants can be identified during the aforementioned law-making process (Biegelbauer and Griessler 2009: 67ff.). This paper focuses on the regulation of access, which refers to the inclusion and exclusion of actors. The involvement of actors typically occurs in a concentric movement, where the responsible ministry is at the centre and moving outwards, others are included over time (Biegelbauer and Griessler 2009; Griessler and Lehner 2010).

The most important phases of the Austrian law-making process are informal. Civil servants collect first comments within their own ministry; then they include other ministries. Thereafter, organizations of interest, most importantly the social partners, are involved although this often occurs with considerable delay. Then, if at all, political parties and less important organizations of interest are asked to comment. In this phase, it is important to mitigate as many potential conflicts of interest as possible. As a matter of routine, civil servants try to exclude the broader public from this pre-consultation phase. This enables privileged actors to communicate and to bargain without losing face. Since Austria is a small country, the fact that there are only a small number of actors, who also tend to know each other, facilitates informality. The result of the informal negotiation process, the ministerial draft, is published and sent out for official comments in the formal consultation phase.

In Austria, civil servants play a much more significant role in law-making than is commonly perceived by social scientists and the broader public. They are not merely subordinates executing orders but are instead influential actors and gate-keepers, who organize, facilitate, negotiate, record and partly co-determine the law-making process. They provide technical expertise and are responsible for details, for example, to negotiate or to formulate a law. They often make preliminary decisions that have to be approved by their political superiors. Ministers, on the other hand, reserve their right to make a decision on a regulation at any time during the decision-making process and enjoy a considerable freedom from the obligation to follow a fixed procedure. The contribution of civil servants to law-making is not restricted to the pre-parliamentarian realm but extends into parliament, for example, into meetings of parliamentary factions or in committee work, where they explain the governmental bill to Members of Parliament. For cases, in which changes of governmental bills are required in the National Council, civil servants make the necessary amendments literally “until the last minute, often even in Parliament” (Griessler and Lehner 2010: 125).

### Neo-corporatism

Austrian policy-making is strongly influenced by neo-corporatism (Pelinka 2008; Tàlos and Kittel 2001), which in the context of law-making refers to the participation of well-organized and well-established interest groups in the pre-consultation and consultation phases. The most important actors are the so-called social partners, a small and closed circle of organizations representing employers and employees. Glynn et al. draw the image of an exclusive club that “is small, the barriers to entry are high and the turnover of leading policy-makers low” (2003: 28). Austrian science and technology policy is also characterized by neo-corporatism. Decisions are typically made by a rather small number of actors from politics, civil service, science and interest organizations (Griessler 2010: 176).

The other side of the coin of neo-corporatism is the limited direct role of the public in policy-making. Austrian civil society is weak and, with the exception of general elections and a few possibilities of direct democracy, citizens are not directly included in policy-making, but only indirectly and pro forma by “obligatory membership in chambers of commerce, labor and agriculture” (Glynn et al. 2003: 29). In recent years, changes towards a strengthening of civil society have happened although they are progressing rather slowly.

Austrian neo-corporatism has to be understood against the background of a long history of a paternalistic relationship between the state and the public, which is condensed in the term “enlightened absolutism” *(Aufgeklärter Absolutismus)*; this refers to a central and formative period of Austrian state, bureaucracy and policy-making in the eighteenth century (Hanisch 2005: 26). Degelsegger and Torgersen describe the tradition of enlightened absolutism, which still exists in Austria today, as benevolently enlightened but basically autocratic. It entails that “policy-makers aim to inform a public supposedly unaware of its own best interests, while they fear obstructive mobilization against their own, in their view, essential function to drive forward objectively necessary, sensible and useful policy projects, balancing powerful interests” (2011: 392).

### Expert orientation

While scientific advice is hardly institutionalized in Austria and mostly occurs “on an irregular and informal basis” (Kronberger and Kerbe 2010: 21), scientists do have an important influence on Austrian politics. However, the direction and extent of this influence are not always clear and foreseeable. Therefore, “it is hard to predict when scientific advice will be followed and when it will not” (ibid.: 22). In the area of biomedicine, scientific experts, particularly physicians, have a dominant power of definition (Griessler 2010). Experts and their established, self-confident and powerful interest lobbying organizations are successfully integrated into Austrian policy-making. In biomedicine and bioethics, a small number of scientific experts and physicians play an important role in committees and advisory boards, such as the Austrian Bioethics Commission, the National Health Council, the Gene Technology Commission and its sub-committees.

### Closeness

In Austria, political practices of law-making are typically elitist and remain behind closed doors. Central processes such as those of the pre-consultation and the consultation phases, as well as parliamentary committee meetings, exclude the public. The plenary session in Parliament, in which the law is put to a vote, is typically the very first debate the public has access to. This session, however, is strongly ritualistic and tends to be a theatrical performance; it is staged for an imagined audience of voters that merely exists in front of a television set. In addition, no decisions are actually made during this debate, apart from a formal acceptance of what was already decided on a long time ago in the pre-parliamentarian phase by powerful actors who are not necessarily present in parliament.

### Taboo

The tendency to avoid and regard political conflict as taboo is an important aspect of Austrian policy-making. Observing taboos avoids entering into strong and seemingly intractable conflicts. The most important taboos in Austrian science and technology policy concern nuclear energy, GMO and—in the area of bio-, and reproductive medicine—the regulation of abortion.

The conflict over the criminal liability of abortion is still influential in Austrian life, science and policies; it continues to affect areas such as human embryonic stem cell research, PND, PGD and in vitro fertilization. It seemingly ended in 1975 with a fragile but lasting compromise that combines permissive legal regulation with partly restrictive implementation (Griessler 2007). The lesson that most policy-makers learned from this conflict was to avoid debates that might disturb this fragile cease-fire (Griessler 2010).

A second major taboo in Austrian science and technology policy is nuclear energy. This conflict ended in 1978 with the decision to terminate the operation of a completed power plant, after the electorate opposed nuclear energy in a popular vote.

A third taboo concerns GMOs, particularly in agriculture. In 1998, the Austrian Government faced a public initiative on gene technology *(Gentechnikvolksbegehren)*, which was extremely critical of GMOs. It demanded restrictive governmental policies and became one of the most successful public initiatives in Austrian history. This success shocked Austrian policy-makers who perceived biotechnology as a means for economic growth. Ever since, policy-makers have aimed to avoid a repeat of the political disaster they stumbled into in the late 1990s.

These conflicts constitute pivotal events in the elite perception of the public in questions of science and technology. All of them were extremely emotional, characterized by seemingly irreconcilable cleavages within society and followed by a political deadlock. They were rare cases in which strong public mobilization challenged the normal method of top-down policy-making, which can be characterized as a “hierarchical techno-political system” (Kronberger/Kerbe: 19).

### Public

#### Policy-makers’ perception of pTA

According to Degelsegger/Torgersen, Austrian policy-makers have a rather hesitant and ambivalent relationship towards pTA; having been confronted with a public that strongly opposed new technologies in the past, they perceive a “tension between the fear of a mobilized public and the need to seek public support” (2011: 396). Policy-makers see a dilemma between the need for public backing and their intention to keep the public away from political discussion and decision making because it could obstruct the introduction of new technologies. There is a fear that pTA might strengthen negative mobilization against technologies (ibid. 396). For these reasons, Austrian policy-makers avoid giving pTA a role to “encourage discussion“(Kronberger and Kerbe 2010: 19), to further participation in policy-making and to increase democratic legitimacy by furthering the “inclusion of alternative rationalities” (Degelsegger and Torgersen 2011: 398). In contrast, they perceive pTA as a means “to inform and convince” the public (Degelsegger and Torgersen 2011: 398).

#### Self-perception of policy-makers and their perception of the public

Austrian policy-makers, experts and representatives of interest groups perceive the public in pronouncedly paternalistic terms (Degelsegger and Torgersen 2011). This is expressed in various ways; firstly, in that they regard themselves as being able to find optimal solutions that they then want to be implemented “with the least possible opposition” (ibid. 400). From this perspective, “it appears impossible (…) that the combined wisdom of the policy, technical and financial experts and the balance of powerful interests could be trumped by lay insights” (ibid.). Secondly, they perceive the public as being “ignorant” (ibid.). Policy-makers believe to “know what the public ‘really’ wants” (ibid. 398) and that they have to act on behalf of the public. Participation, in this line of argument, is intrinsically pointless. In line with this, the relationship between science and the public is characterized by a deficit model, which assumes that the public requires information and education *(Aufklärungsdiskurs,* Felt et al. 2006: 104).

### pTA in the context of Austrian policy-making

In contrast to democracy theory and the widespread public image, the Austrian Parliament—like in many western democracies—actually has a less strong position in law-making than government. This is caused by a lack of financial resources, personnel and expertise. Parliament is less a place of decision making, than an obligatory passage point. Draft bills typically enter Parliament as governmental bills and often leave it almost unchanged.

Since the executive branch has a particularly strong role in law-making, it is necessary to look closely at connected processes and actors. Ministers (and their staff) want to retain the space for political manoeuvre and reserve the power to make political decisions at any time of the political process. Civil servants play a particularly important role in law-making because of their technical expertise; they organize the process and put the law down in writing. Their influence is not limited to the pre-parliamentarian phase, but extends into Parliament. Social partners and political actors of organized interests are also important groups of actors. Within the law-making process, the pre-parliamentarian phase is of utmost importance. It is characterized by neo-corporatism, expert orientation, informality and deliberate exclusion of un-invited actors and the public.

The strong notion of neo-corporatism is also important in science and technology policy. The paternalistic tendency of this policy approach is deeply rooted in Austrian history, for example, in Counter Reformation and in eighteenth century enlightened absolutism. In this tradition, a dominant paternalist elite of policy-makers and experts is sceptical of the public’s ability to form its own sensible political opinion. Nevertheless, public support is considered indispensable to advancing technologies, which are in turn indispensable from the elite’s perspective (Degelsegger and Torgersen 2011).

Issues of science and technology policy are not frequently publicly debated in Austria because of a strong notion of taboo when it comes to controversies and a tendency to procrastinate when it comes to making decisions in divisive areas. Past conflicts over nuclear power, GMOs and abortion cast a cloud over current discussions of science and technology policy (Griessler 2010).

Due to these characteristics of Austrian policy-making, the chances of pTA as a means to actively involve the public in policy-making have to be assessed rather sceptically. In many ways, pTA virtually contradicts central practices and assumptions of Austrian policy-making. So far, pTA has been used in Austria often as “laboratory experiments” by social scientists (Bogner 2010) and is perceived by policy-makers a marketing tool rather than as an instrument to promote public participation.

## Conclusion

The comparison of Switzerland and Austria demonstrates different approaches to xenotransplantation policy-making as well as different applications of pTA.

Switzerland proactively and independently developed its own xenotransplantation policies. It witnessed a small, but informed public debate and a thorough discussion in Parliament. The policy-making process involved many political actors, ministries, parliament, experts, NGOs and was, among other information, also based on TA and pTA. Austria, in contrast, adopted a wait-and-see position and only years later followed European legislation. There was no public debate, discussion in Parliament or expert bodies. The executive, namely civil servants, dealt with the topic and kept it within their realm.

Experiences with pTA were also fundamentally different between Switzerland and Austria. pTA seems to fit the Swiss way of policy-making much better than the Austrian. Despite the similarities between the two small western democracies and although both countries are concordance democracies, there are remarkable differences between the two that distinguish them in terms of being prepared for participatory exercises. Whereas Swiss concordance democracy emphasizes pluralism and a higher distribution of power between a larger number of actors, Austrian concordance democracy is characterized by a dominance of a small number of organized actors which try to pervade society and involve citizens by means of mandatory membership. The most striking and important difference between Switzerland and Austria, however, is the veto power of its citizenry, which is particularly strong in Switzerland but remarkably weak in Austria. In the Swiss context, pTA seems functional to avoid potential resistance from the public. Unlike Austria, there is an emphasis in Swiss policy-making on including minorities. Moreover, there is an emphasis on rationality and matter-of-factness in public discourse, which is contrasted in Austria by a strong role of taboo that is meant to avoid the controversial and emotional debates experienced in the past. There are also differences between the two countries regarding the dominant formative group of society. Whereas in Switzerland the idea of an independent citizenry in policy-making is a powerful image of self-perception, in Austria it is state bureaucracy with its roots in enlightened absolutism, in which citizens are perceived as subjects to be guided.

The two cases of Switzerland and Austria strongly indicate that pTA can only be transferred across countries skilfully and with great caution. pTA has not only to fit into long-standing practices of policy-making but also accord to the self-perception and roles of important actors such as politicians, civil servants, experts, stakeholders, NGOs and the public. This does not at all mean that change towards more participation in science and technology policy is impossible, but that it needs realism, persistence and time.

## Footnotes

1 In the text, interviews are for the most part paraphrased, sometimes they are also quoted verbatim. In both cases, the number of the interview and the line numbers of the transcripts are indicated in the text. Interviews with Swiss interviewees are coded numerically, transcripts with Austrian interviewees alphanumerically.

2 I will use in the following a rough English translation of this name, that is, “Basel Appeal against Genetic Engineering” or short “Basel Appeal”.
